# A Review of Extraintestinal Manifestations & Medication-Induced Myocarditis and Pericarditis in Pediatric Inflammatory Bowel Disease

**DOI:** 10.7759/cureus.26366

**Published:** 2022-06-27

**Authors:** Kevin Cesa, Catherine Cunningham, Tyler Harris, Whitney Sunseri

**Affiliations:** 1 Pediatric Gastroenterology, Hepatology, and Nutrition, University of Pittsburgh Medical Center (UPMC) Children's Hospital of Pittsburgh (CHP), Pittsburgh, USA; 2 Pediatrics, University of Pittsburgh School of Medicine, Pittsburgh, USA; 3 Pediatric Cardiology, University of Pittsburgh Medical Center (UPMC) Children's Hospital of Pittsburgh (CHP), Pittsburgh, USA

**Keywords:** extrainintestinal manifestations, pediatrics, ibd, myocarditis, pericarditis

## Abstract

Inflammatory bowel disease (IBD) is a systemic disorder where extraintestinal symptoms may involve virtually any organ system. Of these extraintestinal symptoms, those involving the heart are relatively rare but associated with significant morbidity. We reviewed the existing literature on noninfectious myocarditis and pericarditis in the pediatric IBD population, including extraintestinal manifestations (EIMs) of IBD and extraintestinal complications (EICs) from medication. We focused on the incidence, presentation, diagnosis, treatment, and outcomes for timely diagnosis and management of these potentially deadly diseases. In addition, we aim to identify and highlight the gaps in current knowledge for future studies and investigations.

## Introduction and background

Extraintestinal symptoms of inflammatory bowel disease

Inflammatory bowel disease (IBD), which includes Crohn’s disease (CD) and ulcerative colitis (UC), is a chronic inflammatory disease that primarily affects the gastrointestinal tract, but extraintestinal symptoms (EISs) can extend to virtually all body systems. The EISs can be divided into two groups: extraintestinal complications (EICs), and extraintestinal manifestations (EIMs) [1]. The EICs arise from the treatment of IBD or due to IBD itself and include drug-related side effects, osteoporosis, nephrolithiasis, and micronutrient deficiency [2,3].

Despite EIMs occurring in approximately 50% of patients, there remains a lack of consensus on the definition, diagnosis, and treatment of these entities [4,5]. In 2019, the European Crohn’s and Colitis Organization suggested the mechanistic definition of EIM to be “an inflammatory pathology in a patient with IBD that is located outside the gut and for which the pathogenesis is either dependent on extension/translocation of immune responses from the intestine or is an independent inflammatory event perpetuated by IBD or that shares a common environmental or genetic predisposition with IBD” [6]. The EIMs can be divided into three groups: EIMs directly related to bowel disease activity such as erythema nodosum, episcleritis, and oral aphthous ulcers; those unrelated to bowel disease activity like ankylosing spondylitis and uveitis; and those with an unclear relationship to bowel disease activity like pyoderma gangrenosum and primary sclerosing cholangitis.

The EISs of IBD are challenging conditions that can adversely impact a patient's quality of life and require a multi-disciplinary team for evaluation and management [7]. Cardiovascular symptoms of IBD, including pericarditis, myocarditis, thromboembolism, arrhythmias, infective endocarditis, and Takayasu arteritis, are rare. The prevalence is estimated to be <1% of patients with IBD [2].

The goal of this paper is to review the existing literature on myocardial and pericardial disease in the pediatric IBD population in a multidisciplinary manner amongst gastroenterologists, cardiologists, and general pediatricians to improve the care of pediatric patients with IBD who develop carditis. In addition, we aim to identify and highlight the gaps in current knowledge to guide future studies and investigations.

Background on myocarditis and pericarditis

Myocarditis refers to inflammation of the myocardium and pericarditis refers to inflammation of the pericardial sac. Pericarditis and myocarditis can occur in concert, coined as myopericarditis or perimyocarditis depending on the predominant location of the inflammation. Pleuropericarditis refers to a case in which there is inflammation of the pericardium and the pleura [8]. The overall incidence rates of pediatric myocarditis and pericarditis are estimated to be 1.95 and 3.32 per 100,000 person-years [9,10]. This is lower compared to the incidence in adults, but the exact incidence is difficult to estimate due to limited available epidemiological data and mild subclinical cases [10,11]. 

Myocarditis is typically caused by infections but may also be caused by autoimmune disease, hypersensitivity reactions, and toxins. Viral causes include enteroviruses, influenza, adenovirus, parvovirus B19, human herpesvirus 6, hepatitis C, Epstein-Barr virus, and cytomegalovirus. Less common infections are caused by bacteria (including *Corynebacterium diphtheria*, *Mycobacterium tuberculosis*, rickettsiae), fungi, helminths, and *Trypanosoma cruzi*. Pericarditis is caused by similar viral and bacterial infections, autoimmune disease, neoplasms, cardiac surgery, trauma, metabolic derangements including hypothyroidism and uremia, and drugs [12].

## Review

Prevalence and etiology of pericarditis and myocarditis in IBD

We identified a total of 28 cases of pediatric patients with IBD with noninfectious myocarditis or pericarditis in the literature (Table 1). Of these 28 cases, 18 were attributed to EICs from the patient’s medication, and 10 were thought to be EIMs of their IBD. Of the 18 case reports of medication-induced carditis: 39% (7/18) were cases of pericarditis, 22% (4/18) of pleuropericarditis, 22% (4/18) of myopericarditis, and 17% (3/18) was a case of myocarditis. Sixteen cases (89%) were secondary to 5-aminosalicylates (5-ASA), including mesalamine and sulfasalazine, and two cases (11%) were secondary to infliximab (IFX), a tumor necrosis factor-alpha (TNF-α) inhibitor.

**Table 1:** Cases of carditis in pediatric patients with inflammatory bowel disease UC: Ulcerative colitis, CD: Crohn’s disease, 5-ASA: 5-aminosalicylic acid,  EIM: Extraintestinal manifestations, YO: Year-old

Case number	Title	First author (year published)	Patient age /gender	IBD phenotype	Carditis phenotype	Proposed mechanism
1	Pericarditis during inflammatory bowel diseases. Extra-intestinal or iatrogenic complication? [13]	Heresbach, D (1994)	17 YO/?	UC	Pericarditis	Medication (mesalamine)
2	Massive pericardial effusion in a child following the administration of mesalamine [14]	Kaiser, G (1997)	9 YO/F	UC	Pericarditis	Medication (mesalamine)
3	Recurrent pericarditis due to mesalamine hypersensitivity: a pediatric case report and review of the literature [15]	Sentongo, T (1998)	16 YO/ M	UC	Pleuropericarditis	Medication (mesalamine)
4	Acute pericarditis associated with 5-aminosalicylic acid (5-ASA) treatment for severe active ulcerative colitis [16]	Ishikawa, N (2001)	17 YO/ M	UC	Pericarditis	Medication (5-asa)
5	Recurrent pericarditis in children and adolescents: report of 15 cases [17]	Raatikka, M (2003)	6 YO/ F	UC	Pleuropericarditis	Medication (mesalamine)
6	Severe chest pain in a pediatric ulcerative colitis patient after 5-aminosalicylic acid therapy [18]	Atay, O (2008)	12 YO/ M	UC	Pericarditis	Medication (mesalamine)
7	Mesalamine-induced myocarditis and coronary vasculitis in a pediatric ulcerative colitis patient: a case report [19]	Perez-Colon, E (2011)	16 YO/M	UC	Myocarditis	Medication (mesalamine)
8	Mesalamine-induced myocarditis following diagnosis of Crohn’s disease: A case report [20]	Braga, C (2013)	19 YO/ M	CD	Myocarditis	Medication (mesalamine)
9	Chest pain in a 12-year-old girl with ulcerative colitis after therapy with mesalazine [21]	Mukherjee, N (2013)	12 YO/ F	UC	Pericarditis	Medication (mesalamine)
10	Mesalamine-induced myopericarditis in a paediatric patient with Crohn’s disease [22]	Nair, A (2015)	17 YO/M	CD	Myopericarditis	Medication (mesalamine)
11	Mesalazine-induced pleuropericarditis in a patient with Crohn’s disease [23]	Kiyomatsu, H (2015)	16 YO/ M	CD	Pleuropericarditis	Medication (mesalamine)
12	Mesalazine-Induced myopericarditis in a patient with a recent diagnosis of Crohn’s disease: apropos of a case [24]	Sorleto, M (2015)	18 YO/M	CD	Myopericarditis	Medication (mesalamine)
13	Pericarditis during infliximab therapy in paediatric ulcerative colitis [25]	Dipasquale, V (2018)	14 YO/M	UC	Pericarditis	Medication (infliximab)
14	Plasma N-terminal pro-B-type natriuretic peptide (BNP) in mesalazine-induced myopericarditis [26]	Paschalis, T (2019)	16 YO/M	CD	Myopericarditis	Medication (mesalamine)
15	Mesalamine-Induced Myopericarditis: A Case Report and Literature Review [27]	Taha, M (2019)	18 YO/F	UC	Myopericarditis	Medication (mesalamine)
16	A Rare Case of mesalazine-induced acute myocarditis in a 19-Year-Old female with ulcerative colitis [28]	Ali, A (2021)	19 YO/ F	UC	Myocarditis	Medication (mesalamine)
17	Antitumor necrosis factor-alpha (TNF-α) infliximab-Induced pleural effusion and pericarditis [29]	Fonseca, A (2021)	18 YO/M	CD	Pleuropericarditis	Medication (infliximab)
18	5-aminosalicylic acid-induced pericarditis in pediatric Crohn’s disease [30]	Toguchi, Y (2022)	14 YO/M	CD	Pericarditis	Medication (mesalamine)
19	Myopericarditis complicating ulcerative colitis [31]	Mowat, N A (1974)	15 YO/M	UC	Myopericarditis	EIM
20	Pericarditis in association with ulcerative colitis [32]	Levin, E N (1979)	12 YO/F	UC	Pericarditis	EIM
21	Myocarditis in children with inflammatory bowel disease [33]	Frid, C (1986)	19 YO/M	UC	Myocarditis with pleural effusion	EIM
22	Myocarditis in children with inflammatory bowel disease [33]	Frid, C (1986)	11 YO/M	CD	Myocarditis	EIM
23	Pericarditis and ulcerative colitis [34]	Farley, J D (1986)	18 YO/M	UC	Pericarditis	EIM
24	Carditis complicating inflammatory bowel disease in children. Case report and review of the literature [35]	Granot, E (1988)	10 YO/M	UC	Pleuropericarditis	EIM
25	Atlantoaxial subluxation and pericarditis in a child with Crohn's disease [36]	Mahajan, L (2001)	9 YO/M	CD	Pericarditis	EIM
26	Recurrent pericarditis as an extra-intestinal manifestation of ulcerative colitis in a 14-year-old girl [37]	Van Gils, A (2018)	14 YO/F	UC	Pericarditis	EIM
27	Recurrent pericarditis in an adolescent with Crohn's colitis [38]	Das, B (2020)	13 YO/M	CD	Pleuropericarditis	EIM
28	Successful treatment with corticosteroids in an 11-year-old patient with Crohn’s disease and myopericarditis—case report [39]	Ryzko, J (2022)	11 YO/F	CD	Myopericarditis	EIM

The prevalence of pericarditis and myocarditis in patients with IBD is higher compared to the general population. A 2005 University of Manitoba registry study found that patients with IBD had an increased risk of pericarditis compared to controls, with a prevalence ratio of 1.96 to 3.33 [40]. Similarly, a Danish registry study found the risk of myocarditis for patients with IBD to be 4.6 per 100,000 years with a risk ratio of 8.3 for Crohn's disease and 2.6 for ulcerative colitis, compared to the background population [41]. A Finnish registry study found that 0.9% of pediatric patients with myocarditis had previously been diagnosed with IBD [9].

We identified additional cases of pericarditis, myocarditis, or pleuropericarditis induced by medications used in the treatment of pediatric IBD in adult patients with inflammatory diseases including adalimumab [42,43], etanercept [44], azathioprine [45], methotrexate (MTX) [46], and topical rectal mesalamine [47].

We identified 10 nondrug-induced non-infectious causes of myocarditis or pericarditis in pediatric patients with IBD in the literature that were thought likely EIMs of the patient’s IBD. Of these case reports, 40% were cases of pericarditis (4/10), 20% were cases of myocarditis (2/10) 20% were cases of pleuropericarditis (2/10), and 20% were cases of myopericarditis (2/10). This cohort was composed of six patients with UC, 4 patients with CD, seven males, and three females.

Signs and symptoms of pericarditis and myocarditis

Pediatric patients with pericarditis and myocarditis often present with nonspecific symptoms that can range from minor discomfort to cardiovascular collapse, which we observed in the current cohort (see Appendices).

The classic presentation of pericarditis is sharp, pleuritic chest pain that improves by sitting up and leaning forward and worsens with lying down, coughing, and/or deep breathing [48]. In our cohort of patients, the most common presenting symptom in the 11 patients with pericarditis was chest pain (82%), which varied in intensity, location, and severity between patients. Additional complaints included dyspnea (37%), fatigue (18%), and palpitations (9%). Around 73% of patients also presented with fevers, including a 12-year-old female who presented with fever and no other complaints [32]. Physical exam findings suggestive of pericarditis include a pericardial friction rub due to inflamed pericardial layers, but this is only found in a minority of patients. In our cohort, the most common abnormal physical exam findings were tachycardia (64%), friction rub (45%), and muffled heart sounds (27%).

Children with myocarditis also present with a variety of symptoms, ranging from mild flu-like symptoms to overt heart failure, cardiogenic shock, and arrest [49]. In our cohort, the most common signs and symptoms in the five patients with myocarditis were dyspnea (60%), chest pain (60%), fatigue (40%), tachycardia (40%), and fever (20%). Prior studies have suggested that patients with myocarditis and an autoimmune or inflammatory disorder are significantly less likely to present with chest pain compared to individuals with idiopathic myocarditis [50].

The five patients with pleuropericarditis presented with similar complaints including tachycardia (50%), chest pain (50%), dyspnea (33%), and shoulder pain (33%). Finally, the six patients with myopericarditis also presented with vague complaints including chest pain (100%), fever (50%), tachycardia (50%), and friction rub (33%).

Evaluation of pericarditis and myocarditis in IBD

After a detailed history and physical exam, the diagnosis of suspected myocarditis and pericarditis includes labs, electrocardiogram (ECG), chest radiography (CXR), and echocardiography (ECHO). Myocarditis remains a challenging diagnosis since endomyocardial biopsy remains the gold standard, and no other single test or study is reliably specific or sensitive [51].

Laboratory findings in pericarditis and myocarditis are dependent on the etiology and extent of inflammation, where elevations of acute phase reactants and white blood count (WBC) can be seen in infectious, autoimmune, and malignant sources etiologies [51]. In the current cohort, 50% had elevated erythrocyte sedimentation rate (ESR) (14/28), 39% had elevated c-reactive protein (CRP) (11/28), and 18% had elevated WBC (5/28). Cardiac biomarkers (typically troponin and brain natriuretic peptide (BNP)) were sent to assess for myocardial injury and negative values were useful to help exclude a diagnosis of myocarditis [52].

Similarly, a CXR may demonstrate pulmonary edema, pulmonary venous congestion, and pleural effusions found in pericarditis and myocarditis. Cardiomegaly can be seen in pericarditis, especially with large effusion. A normal CXR does not exclude pericarditis or myocarditis, since CXRs in acute myocarditis usually show normal heart size [53], including in the current cohort where a patient with myopericarditis had a normal CXR [31]. 

An ECG can show evidence of pericarditis or myocarditis. The most common abnormalities seen in acute pericarditis are diffuse/non-localizing ST-segment elevation and PR-interval depression. Later in the inflammatory process, there is T-wave inversion followed by recovery after inflammation resolution. The ECGs for patients with myocarditis are also often abnormal, with nonspecific findings including sinus tachycardia, ST-segment and T-wave abnormalities, low-voltage QRS complexes as well as atrial and ventricular arrhythmias [51].

A transthoracic ECHO should be performed in suspected cases of pericarditis and myocarditis. An ECHO can show the pericardial effusion associated with pericarditis but may be normal in cases of minimal or loculated fluids. In pericarditis, cardiac function is usually preserved, while the ECHO for patients with myocarditis often shows ventricular dysfunction and wall motion abnormalities [49]. Subtle abnormalities in function can be demonstrated with myocardial strain imaging on ECHO. An ECHO can suggest cardiac tamponade associated with pericarditis (though tamponade is a clinical diagnosis) and other causes of cardiac dysfunction including anatomic and structural abnormalities.

Endomyocardial biopsy is typically reserved for patients where the prognostic and therapeutic value outweigh the potential risks [54]. Clinicians should evaluate the risk/benefit ratio in cases of carditis in patients with IBD where the etiology is unclear since endomyocardial biopsies have identified viral causes (parvovirus-19) of myocarditis in patients on biologic therapy (IFX) [55].

Cardiac magnetic resonance imaging (MRI) is frequently helpful in both the acute evaluation of possible diagnosis as well as for long-term follow-up and prognostication. An MRI can identify areas of inflammation and scarring within the myocardium, define cardiac function and regional wall motion abnormalities as well as rule out other causes [56]. Strict criteria for the diagnosis of myocarditis have been published [57]. The case report by Taha et al. involved a patient whose echo showed normal left ventricular size, thickness, ejection fraction, systolic function, and diastolic function, but the MRI was diagnostic for pericarditis [27].

Mechanism

There is accumulating evidence that IBD results from an inappropriate inflammatory response to intestinal microbes [58]. In addition, regulatory T cells (Tregs) have been shown to play a vital role in preventing excessive intestinal inflammation, and the disruption of Tregs homeostasis may lead to IBD [59]. Programmed Death-1 (PD-1) is a transmembrane glycoprotein expressed on various activated lymphocytes including T cells and B cells. The PD-1 and its ligands, PD-L1, and PD-L2, provide an inhibitory signal that is necessary for homeostasis between T-cell activation, tolerance, and immune-mediated tissue damage. There is growing evidence that altered function of Tregs via the PD-1 pathway is associated with several human inflammatory conditions including IBD and myocarditis [60]. In mouse models, chronic colitis can be induced via the transfer of CD4+CD45RBhigh T cells from normal Bagg albino (BALB)/c mice into mice with severe combined immunodeficiency (SCID). This colitis can be suppressed when CD4+CD25−PD-1+ T cells are co-transferred into these SCID mice [61,60]. In addition, the intestinal epithelial cells in patients with IBD have been shown to express PD-L1 in higher amounts compared to healthy controls [62]. Finally, PD-1-deficient and PD-L1-knockout mice have been shown to develop autoimmune myocarditis [63-65].

As previously stated, 5-ASA therapy is the leading cause of medication-induced pericarditis and myocarditis in pediatric IBD. However, the pathophysiology is poorly understood. The most commonly cited mechanism is a humoral mediated hypersensitivity reaction with the development of antibodies against 5-ASA along with cross-reactivity to some components of the pericardium or myocardium [14]. Other theorized pathways include similar immunoglobulin E (IgE)-mediated allergic reactions, cell-mediated hypersensitivity reactions, or a direct toxic effect from the drug itself [66]. In addition, due to the spectrum of disease and presentation, it has been theorized that not all patients with 5-ASA cardiac disease are induced via the same pathway. Similarly, the mechanism of IFX-induced cardiac disease is unknown. One postulated theory involves a type 3 hypersensitivity with a serum sickness-like reaction [67].

Diagnosis and treatment of drug-induced carditis

There is a diagnostic dilemma for pediatric patients with IBD in distinguishing drug-induced EIC vs EIM-related pericarditis and myocarditis, due to the rarity of the disease and the lack of consensus for the criteria of diagnosis of EIMs of IBD. 

A 2002 case series of 12 patients with 5-ASA-induced pericarditis reported that signs and symptoms typically present two to four weeks after the introduction of the offending agent, but the onset of symptoms can be delayed secondary to concurrent steroids therapy, which is a common induction in patients with IBD [68]. In the current cohort of patients with 5-ASA-induced carditis, 86% (12/14) of patients developed symptoms within two to four weeks of starting the medication (Table 2). In the case reports by Sentongo et al. and Atay et al., patients developed symptoms of carditis six to seven weeks after initial exposure to mesalamine, but both patients were on concurrent steroids as induction therapy for their IBD [15,18]. Drug-induced lymphocyte stimulation tests have been used as an auxiliary test in the diagnosis of drug allergies [22], but prior studies have shown it has a high specificity, but low sensitivity in the diagnosis of allergy to mesalamine and is thus not commonly performed in clinical practice [69].

**Table 2:** Drug-induced carditis in pediatric IBD UC: Ulcerative colitis, CD: Crohn’s disease, 5-ASA: 5-aminosalicylic acid, YO: Year-old, IFX: Infliximab

Case number	Patient age /gender	IBD phenotype	Carditis	Offending medication	Time from starting medication	Adjunctive treatments	Clinical improvement	New IBD maintenance therapy
1	17 YO/?	UC	Pericarditis	Mesalamine	15 days	X	X	X
2	9 YO/F	UC	Pericarditis	Mesalamine	21 days	Drainage (subxiphoid window)	3 days	Steroids (low dose)
3	16 YO/ M	UC	Pleuropericarditis	Mesalamine	7 weeks	Steroids	"days"	Azathioprine
4	17 YO/ M	UC	Pericarditis	5-ASA	2 weeks	Steroids	x	Steroids (oral/enemas)
5	6 YO/ F	UC	Pleuropericarditis	Mesalamine	X	Steroids	x	x
6	12 YO/ M	UC	Pericarditis	Mesalamine	1 week	Methotrexate	1 day	Methotrexate
7	16 YO/M	UC	Myocarditis	Mesalamine	4 weeks	X	1-2 days	x
8	19 YO/ M	CD	Myocarditis	Mesalamine	2 weeks	Aspirin, ACE inhibitors, beta-blockers	“rapid”	X
9	12 YO/ F	UC	Pericarditis	Mesalamine	X	Steroids. drainage (pericardiocentesis)	5 days	Azathioprine
10	17 YO/M	CD	Myopericarditis	Mesalamine	4 weeks	X	1 day	x
11	16 YO/ M	CD	Pleuropericarditis	Mesalamine	19 days	X	1 day	Infliximab
12	18 YO/M	CD	Myopericarditis	Mesalamine	6 weeks	Metamizole steroids	1-2 days	X
13	14 YO/M	UC	Pericarditis	Infliximab	2 weeks	Steroids, ceftriaxone	"days"	x
14	16 YO /M	CD	Myopericarditis	Mesalamine	2 weeks	ACE inhibitors, beta-blockers	4 days	Azathioprine
15	18 YO/F	UC	Myopericarditis	Mesalamine	2 weeks	X	1-2 days	
16	19 YO/ F	UC	Myocarditis	Mesalamine	4 weeks	Inotropes, furosemide	“rapid”	Infliximab
17	18 YO/M	CD	Pleuropericarditis	IFX	12 weeks	Thoracentesis, azithromycin, colchicine (2 months), steroids (2 months)	4 weeks	Ustekinumab
18	14 YO/M	CD	Pericarditis	5-ASA	2 weeks	Pericardial drainage	1-2 days	X

In the current cohort, all 16 pediatric patients with 5-ASA-induced myocarditis or pericarditis had a full recovery (as seen above in Table 2). Five of the patients recovered only after discontinuation of the causative medication, with an improvement of symptoms as quick as 24 hours and normalization of cardiac biomarkers within three days. This included patients with each phenotype of cardiac toxicity: pericarditis, myocarditis, myopericarditis, and pleuropericarditis. Five patients were treated with adjunctive steroid therapy. To date, there are no randomized clinical trials or observational studies that compare withholding medication vs trial of steroids for treatment of 5-ASA-induced pericarditis [66]. Other medical therapies include cardioprotective agents (beta-blockers, ACE-inhibitors), anti-inflammatories (aspirin, metamizole), and MTX (note: it is unclear if this patient was given MTX as a new maintenance medication for IBD or for treatment of the pericarditis). Four patients with pericarditis required drainage either via a subxiphoid pericardial window or pericardiocentesis. While there were no fatalities in this cohort, there is a case report of mesalamine-associated myocarditis in an adult patient who died after they developed ventricular fibrillation and cardiogenic shock [70].

There were two cases of carditis proposed to be secondary to IFX [25,29]. In the case report by Fonseca et al., the patient developed symptoms of pleuropericarditis 12 weeks after initial IFX exposure, but 24 hours after the most recent infusion. The temporal relationship with IFX-induced carditis varies with case reports of patients developing symptoms ranging from 12 days to 12 months after initial drug exposure [71,72]. Prior case reports have argued patients with IFX-induced carditis should be managed with immediate discontinuation of IFX and treatment with steroids [73], which occurred in both patients in the current cohort. There was variability in patients’ clinical improvement, whereas in the study by Dipasquale et al., patients saw improvement after several days, and patients in the study by Fonseca et al. required two months of treatment with colchicine and steroids due to persistent symptoms. Before the availability of biologic therapy for IBD, there was a desire for clinicians to restart 5-ASA as maintenance therapy for IBD. In that cohort, two patients with 5-ASA cardiac toxicity were attempted to restart either on a low dose of the same medication [16] or switch within the class from mesalamine to sulfasalazine [14]. But both had a recurrence of symptoms within six hours of starting the medication. In addition, there was one patient who had five relapses of pericarditis that resolved once the patient’s mesalamine was withheld [17].

In this cohort, patients previously treated with 5-ASA have successfully had their IBD maintained on immunomodulators (azathioprine/MTX) and biologics (IFX). The patient with pleuropericarditis secondary to IFX was switched to vedolizumab and had no recurrence.

Diagnosis and treatment of EIMs pericarditis/myocarditis

Pericarditis and myocarditis secondary to IBD is a diagnosis of exclusion, and other etiologies including infectious and medication-induced must be considered, worked up, and excluded [68].

About 50% of patients (5/10) developed carditis within one month of diagnosis of their IBD. There was a wide range from three years before to seven years post-diagnosis of patients with IBD. This is not uncommon, especially with prior studies suggesting approximately 25% of patients with EIMs develop symptoms before their diagnosis of IBD [74]. The relationship between IBD activity and the development of pericarditis or myocarditis remains unclear. For patients in the current cohort with nondrug-induced pericarditis or myocarditis, 50% (5/10) of patients had active bowel disease during at least one episode of pericarditis or myocarditis. 

Treatment of IBD-associated pericarditis and myocarditis is based on the relief of symptoms, decreasing inflammation, and the prevention of recurrence. Around 90% (9/10) of patients in the current cohort were treated with steroids (Table 3), including four patients who had improved with steroid monotherapy. One patient with pleuropericarditis was successfully treated with aspirin and a pleural tap [35]. The authors of this paper suggest that a trial of non‐steroidal anti‐inflammatory drugs (NSAID) (aspirin or indomethacin) should be considered prior to systematic steroids. Other authors have argued that corticosteroids should remain first-line therapy due to case reports of NSAID monotherapy being insufficient as first-line therapy and the association of NSAIDs with IBD flares [17,37,75]. Additional adjunctive medical therapy during patients’ initial episodes included lidocaine, antiarrhythmics, and sulfasalazine. Three patients required surgical drainage including pleural taps and pericardiocentesis. 

**Table 3:** Carditis as an extra-intestinal manifestation in pediatric IBD UC: Ulcerative colitis, CD: Crohn’s disease, YO: Year-old, IBD: Inflammatory bowel disease

Case number	Patient age /gender	IBD phenotype	Carditis phenotype	Time since IBD diagnosis	Relapse carditis (# of episodes)	Active IBD with carditis	Treatment initial episode	Relapse treatment
19	15 YO/M	UC	Myopericarditis	At diagnosis	Yes (3)	Yes (3 of 4 episodes)	Steroids	Steroids, sulfasalazine
20	12 YO/F	UC	Pericarditis	At diagnosis	Yes (2)	Yes (1 of 3 episodes)	Steroids	Steroids, indomethacin
21	19 YO/M	UC	Myocarditis	7 years post-diagnosis	No	No	Steroids, lidocaine, antiarrhythmics, pleural taps	x
22	11 YO/M	CD	Myocarditis	3.5 years prior to diagnosis	No	No	Steroids	x
23	18 YO/M	UC	Pericarditis	At diagnosis	Yes (1)	Yes (1 of 2 episodes)	Steroids, sulfasalazine	Steroids
24	10 YO/M	UC	Pleuropericarditis	6 months post-diagnosis	No	No	Pleural tap, aspirin	x
25	9 YO/M	CD	Pericarditis	1 month post-diagnosis	No	Yes	Steroids, cefazolin, held mesalamine	x
26	14 YO/F	UC	Pericarditis	2 months post-diagnosis	Yes (1)	Yes (1 of 2)	steroids. dc mesalamine	Steroids, colchicine, sulfasalazine
27	13 YO/M	CD	Pleuropericarditis	4 years post-diagnosis	Yes (4)	No	steroids, pericardiocentesis	Steroids, ibuprofen, colchicine
28	11 YO/F	CD	Myopericarditis	2 weeks	No	No	Steroids, antibiotics, propanol	X

There were no cases of steroid-refractory myocarditis or pericarditis in the current cohort of pediatric patients with IBD. There is a case report of an adult patient who developed acute fulminant myocarditis requiring extracorporeal membrane oxygenation (ECMO) during an exacerbation of UC that was treated with corticosteroids with no improvement in her myocarditis or underlying IBD. The patient’s systemic inflammation was treated with IFX and she had rapid resolution of colitis symptoms and myocarditis [76]. Small case series have suggested that IFX may be an effective treatment for corticosteroid refractory immune checkpoint inhibitor myocarditis, but larger studies are needed to confirm these findings [77,78].

Recurrence of pericarditis is common in children, with reports ranging from 15% to 40% [79]. Inflammatory bowel disease was shown in a Finnish registry study to be an independent predictor of recurrent myocarditis with a hazard ratio of 7.4 [80]. Reoccurrence in the current cohort occurred in 50% (5/10) of the patients. There was an interesting relationship between patients who had clinically active disease and reoccurrence of their IBD, where 80% of those patients (4/5) who had active IBD with an episode of carditis had recurrent episodes of carditis. Conversely, 20% of patients (1/5) without clinically active IBD during their carditis had recurrent episodes of carditis.

A 2013 multicenter, double-blind randomized control trial of adults with acute pericarditis demonstrated that colchicine reduced the rate of symptoms, the number of reoccurrences, and the hospitalization readmission rate, without an increase in adverse events compared to conventional anti-inflammatory therapy with aspirin or ibuprofen [81]. Similarly, a 2015 systematic review, showed adjunctive colchicine was associated with an approximately 50% lower recurrence rate [48]. Two patients with recurrent pericarditis or pleuropericarditis in the current cohort were treated with colchicine. Both patients had resolution of cardiac disease while on treatment, although one patient had a relapse of their pericarditis when the colchicine was weaned, but resolution once colchicine was reintroduced. Diarrhea is a common side effect of colchicine, which can complicate the clinical assessment of patients with IBD [82].

Interleukin-1 receptor antagonist (anakinra) and antibodies (canakinumab) are other emerging therapy for pericarditis. A 2016 a double-blind randomized withdrawal trial with anakinra or placebo for patients with colchicine-resistant, corticosteroid-dependent pericarditis demonstrated that the use of anakinra reduced the risk of recurrence from 90% in the placebo group to 18.2% in the anakinra group [83]. Anakinra and canakinumab were used in a 29-year-old with UC who had recurrent pericarditis despite dual anti-inflammatory therapy (colchicine and naproxen), but both were discontinued secondary to side effects and recurrence of chest pain [84]. 

## Conclusions

Myocarditis and pericarditis can be both an EIM and EIC in pediatric patients with IBD, with no test that is sensitive or specific to distinguish between the two groups. Due to the rarity and range of clinical presentation, the diagnosis of myocarditis and pericarditis is challenging, and clinicians must maintain a high index of suspicion. 

The most common medication used in pediatric IBD associated with the development of carditis is 5-ASA. Patients typically develop symptoms two to four weeks after starting the offending medication, but symptoms may be delayed if the patient is on concurrent steroids. If there is a concern for drug-induced cardiac disease in pediatric IBD patients, then the presumed causative agent should be held to assess for symptomatic improvement while providing supportive care, monitoring for cardiac complications and progression of their IBD. If a patient with presumed 5-ASA cardiac disease does not have prompt improvement with cessation of medication, other etiologies should be investigated and additional adjunctive therapies such as corticosteroids, NSAIDs, or colchicine should be considered to treat the cardiac disease.

Pericarditis and myocarditis should be considered as a rare EIM of IBD. It remains a diagnosis of exclusion. Other etiologies including infectious and medication-induced must be considered, worked up, and excluded. In episodes of pericarditis or myocarditis thought to be an EIM of IBD, corticosteroids should remain the first line of therapy. Reoccurrence of pericarditis or myocarditis in pediatric IBD is common, and the medical team should consider adjunctive therapy with colchicine or anakinra. 

We advocate for continued reporting of carditis in patients with IBD to improve the diagnosis, clarify the etiology, optimize management, and elucidate patient factors that increase the risk for these complex diseases. 

## Appendices

**Table 4:** Patient's symptoms, exams, and initial evaluations CXR: Chest X-ray, LTT: Lymphoblastic transformation test, EF: Ejection fraction, YO: Year-old, ESR: Erythrocyte sedimentation rate, CRP: C-reactive protein, CK-MB: Creatine kinase-MB, BNP: Brain natriuretic peptide, RF: Rheumatoid factor, ANA: Antinuclear antibody, AHA: Antiheart antibodies, IFA: Immunofluorescent assay, NT-proBNP: N-terminal proB-type natriuretic peptide

Appendix Table 1: Patient's symptoms, exams, and initial evaluations								
Case number	Age/ gender	Carditis phenotype	Presenting symptoms	Physical exam	Notable labs	EKG	CXR	Echo
1	17 YO/?	Pericarditis	x	x	LTT with mesalamine positive	X	X	X
2	9 YO/F	Pericarditis	Chest pain (retrosternal), cough	Fever, tachycardia, muffled heart sounds, friction rub	Elevated ESR	Sinus rhythm	Cardiomegaly	Pericardial effusion
4	17 YO/M	Pericarditis	Chest pain, fatigue	Fever, tachycardia, muffled heart sound	Elevated WBC, CRP, normal troponin T	ST-elevation (limb and precordial leads)	X	Pericardial effusion, left ventricle hypokinesis
6	12 YO/ M	Pericarditis	Chest pain, fatigue	Fever, tachycardia	Normal troponin-T, CK-MB, BNP, CRP	Non-specific ST-T wave changes, T-wave inversion (lateral leads)	X	Pericardial effusion, depressed left ventricular systolic function
9	12 YO/ F	Pericarditis	Chest pain, dyspnea, shoulder pain	Fever, tachycardia, muffled heart sounds, pericardial friction rub	Elevated troponin T	ST-elevation	Cardiomegaly	Global effusion, signs of hemodynamic compromise
13	14 YO/M	Pericarditis	Chest pain (sharp, left-sided)	Tachycardia	Elevated troponin I, CRP, ESR	ST-elevation (global)	X	Mild detachment of pericardium on the right ventricular wall
18	14 YO/M	Pericarditis	Chest pain, dyspnea	Fever, tachycardia	Elevated ESR, CRP, normal troponin T	Diffuse ST-elevation	X	Massive pericardial effusion
20	12 YO/F	Pericarditis	X	Fever, pericardial friction rub	X	ST-elevation, PR depression, T wave inversion,	Cardiomegaly	X
23	18 YO/M	Pericarditis	Chest pain (pleuritic), dyspnea	Fever, systolic murmur, pericardial friction rub	Elevated ESR	X	Cardiomegaly	Pericardial effusion
25	9 YO/M	Pericarditis	Chest Pain, shoulder pain, Sore throat, Neck pain, Cough	Fever, tachycardia, friction rub	Elevated ESR	T-wave abnormalities	Cardiomegaly, pleural thickening	Pericardial effusion
26	14 YO/F	Pericarditis	Chest pain, palpitation	Bouncing pulses	Elevated ESR	Flattened T-waves	X	X
3	16 YO/M	Pleuropericarditis	Chest pain (pleuritic), dyspnea, myalgia	Fever, tachycardia, wheezes (bilateral)	Elevated ESR, normal WBC	X	Cardiomegaly, pulmonary infiltrates, pleural effusion	Pericardial effusion
5	6 YO/ F	Pleuropericarditis	x	x	X	X	X	X
11	16 YO/ M	Pleuropericarditis	Shoulder pain, cough	Fever	Elevated CRP, ESR, positive drug lymphocyte stimulation tests for mesalamine	X	Cardiomegaly, pleural effusion	Pericardial effusion, left ventricle hypokinesis
17	18 YO/M	Pleuropericarditis	Chest pain (pleuritic)	X	Elevated WBC, CRP, RF, ANA, IFA, AHA	Nonspecific ST abnormalities	Blunting of costophrenic angle	Small circumferential pericardial
24	10 YO/M	Pleuropericarditis	Shoulder pain, dyspnea, cervical pain	Tachycardia, pericardial friction rub, tachypnea	Elevated ESR, WBC	Nonspecific ST-T changes, low voltage	Cardiomegaly, pleural effusion	Pericardial effusion, decreased left ventricular function
27	13 YO/ M	Pleuropericarditis	Chest pain	Pericardial friction rub	Elevated CRP	ST-T elevation (diffuse)	Cardiomegaly	Pericardial effusion, normal biventricular systolic function
7	16 YO/M	Myocarditis	Chest pain (throbbing)	Tachycardia, hypertension	Elevated troponin-I	Sinus tachycardia	X	Decreased left ventricular systolic function, left ventricular dilation, coronary ectasia
8	19 YO/ M	Myocarditis	Chest pain (retrosternal, radiating to left arm)	"unremarkable"	Elevated troponin I, CK, NT-proBNP, WBC, CRP	Sinus rhythm and slight ST-segment elevation leads I, II, III, aVF. V3-V6	X	Moderately depressed left ventricular systolic function
16	19 YO/ F	Myocarditis	Chest pain (pleuritic), dyspnea	Tachycardia, hypoxic, hypotensive	Elevated troponin-T	Sinus tachycardia		Dilated cardiomyopathy/ myocarditis w/ severely impaired left ventricular systolic function and EF
21	19 YO/ M	Myocarditis with pleural effusion	Fatigue, dyspnea	X	Elevated ESR	X	X	Pleural effusions, enlarged heart, very little movement
22	11 YO/M	Myocarditis	Fatigue, dyspnea	Fever	Elevated ESR	ST depression	X	X
9	17 YO/M	Myopericarditis	Chest pain (pleuritic), facial numbness	Normal	Elevated troponin 1, CK-MB	ST elevations, T-wave abnormalities	X	Decreased left ventricular systolic function
12	18 YO/M	Myopericarditis	Chest pain (retrosternal)	Normal	Elevated troponin, CK, CRP	Sinus rhythm and ST-segment elevation II, III, aVF, and V4–V6	X	X
14	16 YO /M	Myopericarditis	Chest pain (pleuritic), dyspnea, malaise	Fever, pericardial rub	Elevated troponin T, CRP, p-BMP	Sinus tachycardia	X	Pericardial effusion, left ventricular systolic dysfunction
15	18 YO/F	Myopericarditis	Chest pain (pleuritic), pre-syncope	Tachycardia, flow murmur	Elevated troponin I, CRP, ESR, WBC	Tachycardia borderline T-wave abnormalities in leads II, III, aVF	Bibasilar opacities	Normal left ventricular size, thickness
19	15 YO/ M	Myopericarditis	Chest pain (retrosternal)	Fever, tachycardia, friction rub	Elevated ESR	ST abnormalities, T-wave inversion	Normal	X
28	11 YO/F	Myopericarditis	Chest pain	Fever, tachycardia	Elevated CRP, WBC, troponin, CK-MB	Elevated ST segment V2–V6	X	Pericardial effusion with hyperkinetic circulation
